# Salt as a Medicine

**Published:** 1888-12

**Authors:** 


### SALT AS A MEDICINE.

We are very apt to seek some far fetched cure for our bodily ailments, and to overlook the simpler remedies, quits as affective it may be, which are to be found even in the humblest household.

Common salt is one of the specifics for various diseases, which many lose the benefit of because they are not aware of its value.

For sore or inflamed eyes, wash them in a weak solution of salt and warm water. This is also usefnl to remove the inflammation caused by extraneous substances in the eye.

For sore throat and a hacking cough, take one salt-spoonful of salt, two teaspoonfuls of vinegar, to half a goblet of cold water (iced-water preferable) ; sip this frequently and relief will be felt at once. This same preparation will remove nausea, and settle the weakest stomach. It is also beneficial in attacks of colic.

Nothing is more useful in sickness than a small flannel bag filled with salt. For toothache colic, or any disease requiring warm applications, it is invaluable, as it retains its heat a long time ; and it is greatly to be preferred to hot, wet emolients, which soon get cold and uncomfortable. The bag and all can be put on a tin pan and warmed in the oven ; but it is better to rip a small hole in the bag, and empty the salt out into the pan to heat. After it is hot it can be put back with a large spoon, and the hole sewed up in a moment.

We have recommended this to several of our friends while they were suffering severe pain, and we have had the satisfaction of knowing that they experienced almost immediate relief from it. We knew it prescribed years ago for a case of severe colic. The effect was magical : and ever since the salt-bag has held an honored place in our domestic pharmacy.

A mixture of ice and salt, in proportion of one to one-half, applied to the head frequently gives instant relief from acute headache. It should be tied up in a small linen cloth, like a pad. and held as near as possible to the seat of the pain.

A teaspoonful of salt dissolved in water, and taken every hour or two beginning six or eight hour before a chill, will often prevent it, in intermittent, or what is known as "chills and fever."

I once succeeded with this simple remedy in an obstinate case, where quinine, arsenic, and all the ordinary means had failed.

